# Effects of cord pretension and stiffness of the Dynesys system spacer on the biomechanics of spinal decompression- a finite element study

**DOI:** 10.1186/1471-2474-14-191

**Published:** 2013-06-19

**Authors:** Shih-Liang Shih, Chien-Lin Liu, Li-Ying Huang, Chang-Hung Huang, Chen-Sheng Chen

**Affiliations:** 1 Department of Orthopaedic Surgery, Zhong-Xing Branch of Taipei-City Hospital, Taipei, Taiwan; 2 Institute of Neuroscience, National Chengchi University, Taipei, Taiwan; 3 Department of Orthopaedic Surgery, Taipei-Veterans General Hospital, Taipei, Taiwan; 4 Department of Plastic and Reconstructive Surgery, Chang Gung Meorial hospital, Taipei, Taiwan; 5 Department of Biomedical Research, Mackay Memorial Hospital, Tamshui Taipei County, Taiwan; 6 Department of Physical Therapy and Assistive Technology, National Yang-Ming University, Taipei, Taiwan

**Keywords:** Adjacent disc, Decompression, Dynesys, Cord pretension, Spacer, Finite element analysis

## Abstract

**Background:**

The Dynesys system provides stability for destabilized spines while preserving segmental motion. However, clinical studies have demonstrated that the Dynesys system does not prevent adjacent segment disease. Moreover, biomechanical studies have revealed that the stiffness of the Dynesys system is comparable to rigid fixation. Our previous studies showed that adjusting the cord pretension of the Dynesys system alleviates stress on the adjacent level during flexion. We also demonstrated that altering the stiffness of Dynesys system spacers can alleviate stress on the adjacent level during extension of the intact spine. In the present study, we hypothesized that omitting the cord preload and changing the stiffness of the Dynesys system spacers would abate stress shielding on adjacent spinal segments.

**Methods:**

Finite element models were developed for - intact spine (INT), facetectomy and laminectomy at L3-4 (DEC), intact spine with Dynesys system (IntDyWL), decompressed spine with Dynesys system (DecDyWL), decompressed spine with Dynesys system without cord preload (DecDyNL), and decompressed spine with Dynesys system assembled using spacers that were 0.8 times the standard diameter without cord pretension (DecDyNL0.8). These models were subjected to hybrid control for flexion, extension, axial rotation; and lateral bending.

**Results:**

The greatest decreases in range of motion (ROM) at the L3-4 level occurred for axial rotation and lateral bending in the IntDyWL model and for flexion and extension in the DecDyWL model. The greatest decreases in disc stress occurred for extension and lateral bending in the IntDyWL model and for flexion in the DecDyWL model. The greatest decreases in facet contact force occurred for extension and lateral bending in the DecDyNL model and for axial rotation in the DecDyWL model. The greatest increases in ROMs at L2-3 level occurred for flexion, axial rotation and lateral bending in IntDyWL model and for extension in the DecDyNL model. The greatest increases in disc stress occurred for flexion, axial rotation and lateral bending in the IntDyWL model and for extension in the DecDyNL model. The greatest increases in facet contact force occurred for extension and lateral bending in the DecDyNL model and for axial rotation in the IntDyWL model.

**Conclusions:**

The results reveals that removing the Dynesys system cord pretension attenuates the ROMs, disc stress, and facet joint contact forces at adjacent levels during flexion and axial rotation. Removing cord pretension together with softening spacers abates stress shielding for adjacent segment during four different moments, and it provides enough security while not jeopardizes the stability of spine during axial rotation.

## Background

Surgical treatment for degenerative spinal disorders consists of decompression and spinal column stabilization [1]. Decompression removes hypertrophyic tissues at the diseased spinal segments that can cause spinal canal stenosis and place pressure on the spinal cord or nerve roots. Extensive decompression, however, increases range of motion (ROM) of spinal column [2,3]. Therefore, spine instrumentation is used to stop the motion at painful vertebral segment and facilitate bone fusion.

The pedicle rod system had been the gold standard for spinal stabilization for three decades. This rigid fixation system ensures the fusion of the grafted bones and secures the stability of the bridged level. However, the spinal fusion procedure was found to cause abnormal stress on the adjacent segment. The high stiffness of the pedicle rod system was supposed to be implicated with the role in accelerating degenerative changes at the adjacent levels of the spinal column [4-7].

The Dynesys spinal system (Zimmer Spine, Minneapolis, MN, USA), introduced in 1994, is a pedicle screw-based dynamic stabilization system designed to provide dynamic stability to the spinal column and prevent the acceleration of degeneration caused by spinal fusion procedure [8]. This system can reliably stabilize the spine without bone fusion and has shown good clinical results for degenerative spines [9-13]. However, several clinical studies have reported that the Dynesys system does not prevent the acceleration of degeneration at adjacent spinal levels [9,12,14-16]. The stiffness of the Dynesys system may be an important factor related to degeneration at adjacent levels [17-19].

Schmolez et al. [20] reported that the ROM and neutral zone of the adjacent segments were not affected by the Dynesys instrumentation. They also reported that intradiscal pressure at the bridged level decreased during lateral bending, flexion, and extension although intradiscal pressure at the adjacent levels changed relatively little. Shin et al. [21] used a spring element to simulate the effects of dynamic stabilization devices, and established three models with different dynamic stabilization devices stiffness. The stiffness of the posterior dynamic stabilization device did not affect ROM and intradiscal pressure at adjacent motion segments. Zhang [22] investigated the effects of implant stiffness on disc loading under compression and suggested that the stiffness of the dynamic stabilization device should be lower than 2,000 N/m to provide a bridged annulus stress akin to the intact spine.

In our previous study we examined the effect of different cord preloads on the Dynesys system [23] and revealed that high stiffness was related to high preloads of approximately 300 N on the cord. We also demonstrated that preloads as low as 100 N can reduce facet joint contact forces and disc stress at adjacent levels during flexion. Another study [24] revealed that smaller Dynesys system spacers result in lower stress at adjacent levels during extension, and higher stress during flexion compared to standard size spacers. Diminishing cord preload seems to alleviate adjacent segment disease. However, there is currently no strategy to preserve the stability of the bridged level without concentrated force on the adjacent spinal levels.

To discover how to reduce the stiffness of the Dynesys system without jeopardizing stability, we used finite element (FE) analysis to evaluate the effects of the Dynesys system on the lumbar spine during different moments with and without pretension load. The FE models were simulated degenerative spinal columns that had undergone decompression. ROM, disc stress, and facet joint contact forces were calculated at the bridged level (L3-4) and the adjacent cephalic level (L2-3).

## Methods

### FE model of the lumbar spine and the posterior dynamic stabilization system

We used a validated degenerative FE spinal model consisting of 3-dimensional osseoligamentous L1-L5 vertebrae with a Dynesys system inserted at L3-L4 level. In addition to the osseous vertebrae, the model contained intervertebral discs, endplates, posterior bony elements and all seven ligaments. These intervertebral discs were composed of a ground substance, the annulus fibrosus, and the nucleus pulposus, with 12 double cross-linked fiber layers embedded in the ground substance. The annulus substance was modeled as a hyperelastic material, and the nucleus pulposus was modeled as an incompressible substance because it displayed both solid and liquid viscoelastic charactereristics. To simulate degeneration, the elastic modulus and the Poisson's ratios of the ground substance and the nucleus pulposus were adjusted [24-27] to be in accordance with the study by Umebara et al. [28] that reported an increase in elastic modulus with disc degeneration. The elastic modulus of the ground substance was simulated using a nonlinear hyperelastic, two-parameter (C_10_, C_01_) Mooney-Rivlin solid model. C_10_ and C_01_ represent the constants used with the FE software (ANSYS 10; Swanson Analysis, Houston, PA, USA). These constants characterized the deviatoric deformation according to the Mooney-Rivlin solid model for material constants C_1_ and C_2_ respectively. The elastic modulus was increased in 10% increments by adjusting C_10_ and C_01_ according to an approximate equation for elastic modulus *E* (*E*=6(C_10_+C_01_) [29]. The initial values for C_10_ and C_01_ were 0.42 and 0.105, according to the 3.15 MPa elastic modulus of intact spine reported by Rohlmann et al. [30]. The degenerated disc was validated for each adjustment using FE anaylsis with a motion segment L4-5 model. The ROM of the L4-5 model was compared to those of an in vitro study by Kettler et al. [31]. The modulus for the nucleus pulposus was adjusted according to Ruberte et al. [32] and was validated by applying a 2,000 N compression force at the top of the L4-5 FE model. The disc annulus stress distribution was compared to values reported in an in vitro study by McMillan et al. [33]. The final modified moduli values are listed at Table 1.

**Table 1:** **Material properties of lumbar spine**[20-23]

**Material**	**Element type**	**Young’s modulus (MPa)**	**Poisson’s ratio**	**Area (mm**^**2**^**)**
**Bone**				
Cortical	8-node SOLID185	Ex=11300	ν xy=0.484	-
		Ey=11300	ν xz=0.203	-
		Ez=22000	ν yz=0.203	
		Gx=3800		
		Gy=5400		
		Gz=5400		
Cancellous	8-node SOLID185	Ex=140	ν xy=0.45	-
		Ey=140	ν xz=0.315	
		Ez=200	ν yz=0.315	
		Gx=48.3		
		Gy=48.3		
		Gz=48.3		
Posterior element	8-nodeSOLID185	3500	0.25	-
**Disc**				
Nucleus pulposus	8-node SOLID185	1.66	0.499	-
Ground Substance	8-node SOLID185	5.36	0.45	-
	d=1.12e-007	C_10_=0.42	C_01_=0.105	
**Annulus fibers**	2-node LINK10			
Outermost		550	-	0.76
Second		495	-	0.5928
Third		412.5	-	0.4712
Innermost		357.5	-	0.3572
**Endplate**	8-node SOLID185	24	0.4	-
**Ligament**	2-node LINK10			
ALL		7.8	-	24
PLL		10	-	14.4
TL		10	-	3.6
LF		15	-	40
ISL		10	-	26
SSL		8	-	23
CL		7.5	-	30

C10 and C01 are the two parameters of the Mooney-Rivlin hyperelastic formation; d, material incompressibility parameter, *ALL* Anterior longitudinal ligament, *CL* Capsular ligament, *ISL* Interspinous ligament, *LF* Ligamentum flavum, *PLL* Posterior longitudinal ligament, *SSL* Supraspinous ligament, *TL* Transverse ligament.

### Decompression and stabilization of the lumbar spine

As part of the decompression procedure, a laminectomy was performed by removing the inferior half of the L3 lamina and the superior half of the L4 lamina. The interspinal and supraspinatous ligaments between L3 and L4 were omitted. A facetectomy was performed by removing the medial one fourth portions of the bilateral L3-4 facet joints.

**Figure 1:**
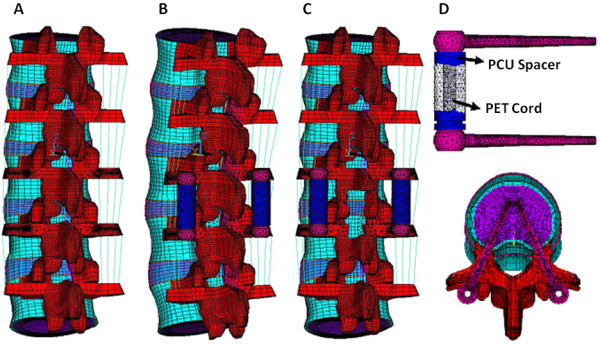
**The finite element model used in this study.** (**A**). The intact spine. (**B**). The intact spine implanted with the Dynesys system in place between L3 and L4. (**C**). The spine after laminectomy and facetectomy between L3 and L4 implanted with the Dynesys system in place. (**D**). The Dynesys system consists of conical titanium alloy pedicle screws, hollow polycarbonate urethane (PCU) spacers and polyethylene terephthalate (PET) cords [20].

The Dynesys system bridging L3-4 was composed of four conical titanium alloy pedicle screws, two hollow polycarbonate urethane (PCU) spacers, and two polyethylene terephthalate (PET) cords. The pedicle screws and the spacers were modeled using eight-node solid elements. The cords were modeled using a two-node tension-only link element. The pedicle screws and the vertebrae were firmly connected to each other. The Dynesys system material properties used in the FE models are detailed in Table 2. Models were also constructed for spacers with diameters of 0.8 times the standard spacers diameters. In total we constructed six models for testing: an intact spine (INT), a spine undergoing laminectomy and facetectomy with resection of the bilateral medial 25% of the facet joints and division of the supraspinous and interspinous ligaments (DEC), an intact spine implanted with the Dynesys system with pretension loading on the cord (IntDyWL), a decompressed spine implanted with the Dynesys system with pretension loading on the cord (DecDyWL), a decompressed spine implanted with the Dynesys system without cord pretension (DecDyNL), and a decompressed spine implanted with the Dynesys system without cord pretension using spacers of 0.8 times the standard diameter (DecDyNL0.8). The Dynesys system and the FE models of the spine with and without the Dynesys system are illustrated in Figure 1.

**Table 2:** **Material properties of the dynesys system**[20-23]

**Components**	**Element type**	**Young's modulus (MPa)**	**Poisson's ratio**
Titanium	8-node SOLID185	110000	0.28
PCU spacer	8-node SOLID185	68.4	0.4
PET cord	8-node SOLID185	1500	0.4

### Boundary and loading conditions

With the lumbar FE spine model constrained at the bottom of the fifth vertebra, the first step of loading was applying 300 N preload to the cords for the IntDyWL and DecDyWL models. The second step of loading was applying to all of the models with 150 N preload on the top of the first vertebra perpendicular to the endplate [34]. The third step of loading was applying four pure moments (i.e., flexion, extension, left axial torsion and left lateral bending) to the top of the vertebrae for all of the spinal models. The hybrid method demonstrated by Panjabi [35] was used to comprehensively evaluate the effects on the adjacent spinal level. Increasing pure moments were applied until the total range of motion reached 20 degrees for flexion and lateral bending, 15 degrees for extension, and 8 degrees for axial rotation.

ROM was calculated at the Dynesys system bridging level (L3-4) and the cranial adjacent level (L2-3) for the four different moments. The von Mises stress for the disc annulus fibrosis and the facet joint contact force at the bridged and cranial adjacent levels were also evaluated.

## Results

### Range of motion at the bridged level

ROM increased the most for the DEC model for all four moments due to hypermobility after decompression. In all of the models implanted with the Dynesys system, the ROM decreased dramatically due to the restricting effect of the implant. The DecDyNL0.8 model showed the smallest decreases in ROM during flexion (-58.96%) and lateral bending (-32.48%) compared to the INT model. The IntDyWL model showed the smallest decrease in ROM during extension (-20.47%), but it displayed the largest decreases in ROM during axial rotation (-12.20%) and lateral bending (-45.87%) compared to the INT model. The largest decreases in ROM from the INT model occurred for the DecDyWL model during flexion (-83.46%) and for the DecDyNL model during extension (-35.91%). It is noteworthy that the DecDyNL model and the DecDyNL0.8 model increased in ROM compared to the INT model (3.90% and 3.41%, respectively) during axial rotation (Table 3).

**Table 3:** **ROM**, **disc annulus stress and facet contact forces on the left** (**L**) **and right** (**R**) **sides of the spine durings flexion**, **extension**, **axial rotation and lateral bending moments**

	**L2**/**3**				**L3**/**4**			
**ROM (Degree)**	**Flexion**	**Extension**	**Rotation**	**Bending**	**Flexion**	**Extension**	**Rotation**	**Bending**
**INT**	4.66	3.42	1.75	4.74	4.8	3.37	2.05	5.08
**DEC**	4.48	3.03	1.75	4.74	5.36	4.7	2.14	5.1
**IntDyWL**	5.88	3.65	1.83	5.4	0.93	2.68	1.8	2.75
**DecDyWL**	5.81	3.65	1.78	5.39	0.89	2.66	1.95	2.9
**DecDyNL**	5.52	3.77	1.76	5.28	1.97	2.16	2.13	3.28
**DecDyNL0**.**8**	5.52	3.7	1.76	5.19	1.97	2.47	2.12	3.43
**Disc stress (KPa)**								
**INT**	998.45	741	332	1316	944.39	687	372	1229.8
**DEC**	946.33	677.64	311.59	1318.4	1047.7	1446	438.63	1292.9
**IntDyWL**	1343	803.04	347	1588	440	592.36	522	793
**DecDyWL**	1323.2	792.27	337.29	1585.7	435.47	618.55	608.98	867.54
**DecDyNL**	1236.2	822.5	324.51	1531.1	558.48	579.08	397.79	780.8
**DecDyNL0**.**8**	1235.9	804.68	324.22	1500.2	558.36	607.8	404.13	859.36
**Facet contact force (N)**								
**INT L**	0.00	86.44	0.00	15.52	0.00	109.61	0.00	26.75
**INT R**	0.00	86.44	117.81	10.29	0.00	109.61	110.68	0.00
**DEC L**	0.00	67.85	0.00	14.15	0.00	77.13	0.00	32.87
**DEC R**	0.00	67.85	102.47	10.48	0.00	77.13	106.24	0.00
**IntDyWL L**	0.00	98.73	0.00	32.76	7.32	65.88	0.00	35.81
**IntDyWL R**	0.00	98.73	124.72	14.77	7.32	65.92	123.06	24.97
**DecDyWL L**	0.00	99.07	0.00	34.20	0.00	21.96	0.00	15.79
**DecDyWL R**	0.00	99.07	120.79	17.32	0.00	21.96	102.52	17.23
**DecDyNL L**	0.00	104.66	0.00	38.18	0.00	13.24	0.00	11.80
**DecDyNL R**	0.00	104.66	112.35	18.34	0.00	13.24	114.98	0.00
**DecDyNL0**.**8 L**	0.00	101.51	0.00	33.71	0.00	15.92	0.00	14.64
**DecDyNL0**.**8 R**	0.00	101.51	112.41	17.47	0.00	15.92	115.14	0.00

***INT*** Intact spine, ***DEC*** Decompressed spine.

***IntDyWL*** Intact spine implanted with the Dynesys system with cord pretension.

***DecDyWL*** Decompressed spine implanted with the Dynesys system with cord pretension.

***DecDyNL*** Decompressed spine implanted with the Dynesys system without cord pretension.

***DecDyNL0.8*** Decompressed spine with Dynesys assembled using 0.8 times diameter spacers wihtout cord pretension.

### Range of motion at the adjacent cranial level

In contrast to the bridged level, the greatest increases in ROM at the adjacent cranial level occurred for the IntDyWL model during flexion (26.18%), left rotation (4.57%) and lateral bending (13.92%) compared to the INT model. The greatest increase in ROM for the DecDyWL model occurred during extension (10.23%). Without the effect of the Dynesys system at L3-4, the ROM for the DEC model increased the least during axial rotation (0%) and lateral bending (0%), and it decreased during flexion (-3.86%) and extension (-11.40%).

### Disc annulus stress at the bridged level

**Figure 2:**
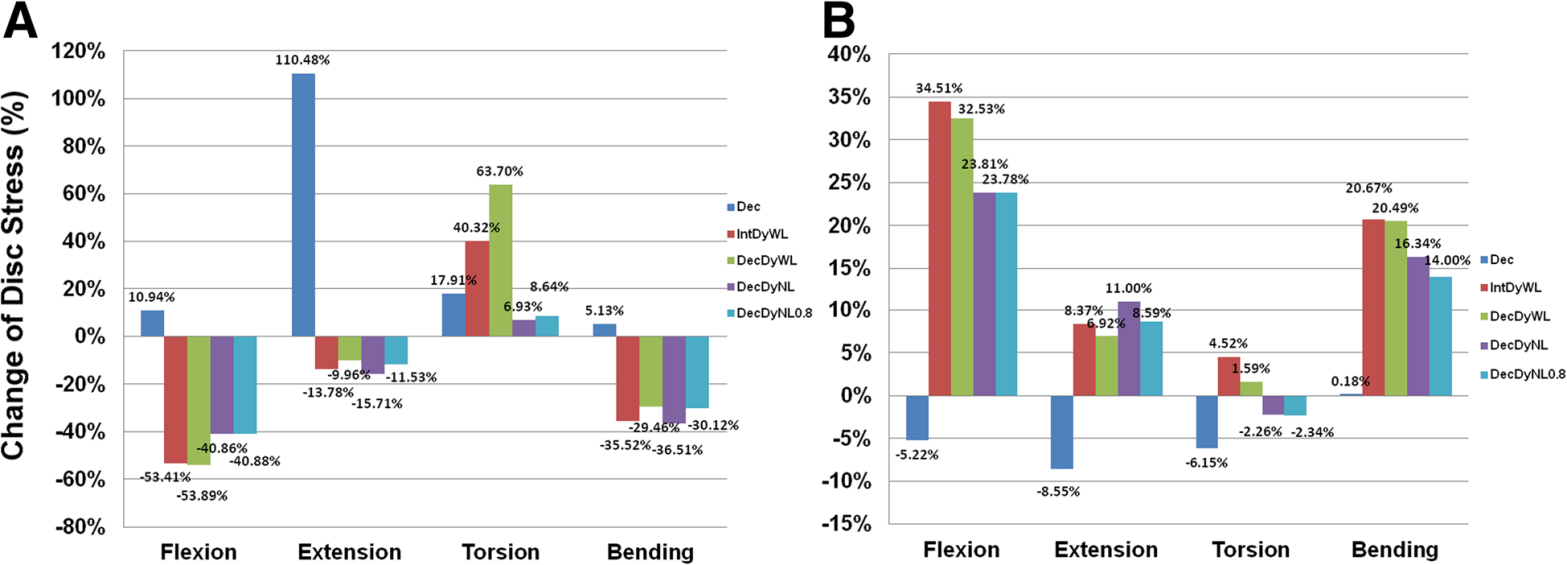
**Disc annulus stress.** Stress at L3-L4 (**A**) and L2-L3 (**B**). Values for the INT are not presented because the data were normalized to the INT with the difference divided by INT values presented as a percentage.

Disc stress for the DEC model increased during flexion, extension, and lateral bending because of decompression at this level. However, for the models implanted with the Dynesys system, the disc stress decreased during the same three moments due to shielding by the Dynesys system. The DecDyNL and DecDyNL0.8 models showed the smallest decrease in disc stress during flexion (-40.86% and -40.88%, respectively, compared with the INT model). The DecDyWL model showed the smallest decrease in disc stress during extension (-9.96%) and lateral bending (-29.46%), and it showed the largest decrease in disc stress during flexion (-53.89%). The DecDyNL showed the largest decrease in disc stress during both extension (-15.71%) and lateral bending (-36.51%), and it showed the smallest increase in disc stress during axial rotation (6.93%). However, the two Dynesys system models loaded with cord pretension (i.e., IntDyWL and DecDyWL) showed greater disc stress than the INT model (40.32% and 63.70%, respectively) during axial rotation (Figure 2A).

### Disc annulus stress at the adjacent cranial level

Compared to the INT model, disc stress at the adjacent cranial level increased for all of the models implanted with the Dynesys system for all four moments, except for the DecDyNL (-2.26%) and DecDyNL0.8 (-2.34%) models during axial rotation. The IntDyWL model showed the greatest increase in disc stress during flexion (34.51%), axial rotation (4.52%) and lateral bending (20.67%), and the DecDyNL model sustained the highest stress during extension (11.00%) compared with the INT model. The DEC models without the Dynesys system at L3-4 showed the smallest increase in disc stress during extension (0.13%) and lateral bending (0.18%), and it showed the largest decrease disc stress during flexion (-5.22%) and axial rotation (-6.15%) at the adjacent cranial level (Figure 2B).

### Facet joint contact force at the bridged level

During flexion, only the IntDyWL model showed sustained facet contact force (7.32 N). For the decompressed spine and spine implanted with the Dynesys system, facet contact force decreased markedly during extension, with the greatest contact force occurring for the DEC (109.61 N) and the smallest contact force occurring for the DecDyNL model (13.24 N). During rotation to the left, only the right facet joints bore contact force. During lateral bending, the left facet joints bore more contact force than the right facet joints. During axial rotation, the IntDyWL model bore the greatest contact force (123.06 N, right side), and the DecDyWL model bore the least contact force (102.52 N, right side). During lateral bending, the IntDyWL model bore the greatest contact force (35.81 N, left side) and the DecDyNL model bore the least contact force (11.8 N, left side) (Table 3).

### Facet joint contact force at the adjacent cranial level

The DEC model sustained the least contact force during extension (67.85 N), axial rotation (102.47 N, right side), and lateral bending (15.14 N, left side). The DecDyNL model bore the most contact force during extension (104.66 N) and lateral bending (18.34 N). The IntDyWL model bore the greatest contact force during axial rotation (124.72 N) (Table 3).

**Figure 3:**
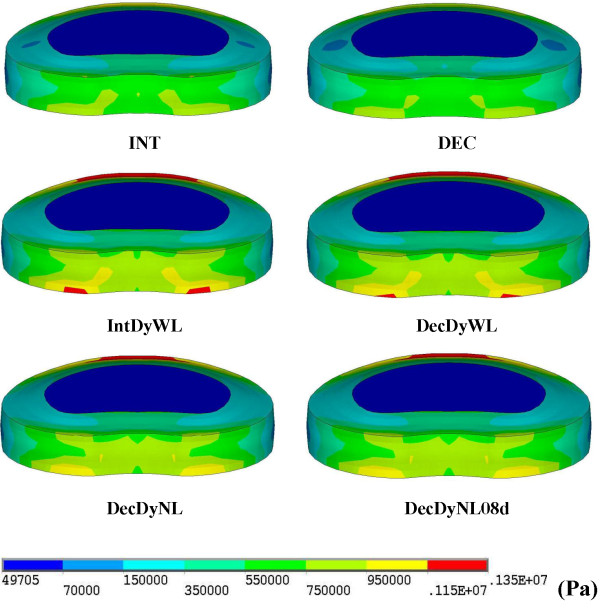
**Disc stress distribution at the adjacent cranial level.** The greatest annulus stress occurred at the anteriosuperior edge of the disc with both lateral sides sustaining less stress. The IntDyWL model showed the greatest annulus stress.

The detail results for ROM, disc annulus stress, and facet contact forces are presented in Table 2. During flexion, the facet joints bore no contact force, thus the discs completely sustaining the stress from the preload and the flexion movement. The von Mises stress at the L2-3 disc annulus illustrated in Figure 3 demonstrated the distribution of stress.

## Discussion

We expected the Dynesys system to provide stability to the destabilized spine and preserve bridged level joint motion. Several clinical reports, however, have shown that adjacent disc disease did not decrease in patients treated implanted with the Dynesys system [9,13,15,16]. Biomechanical studies have shown that disc stress at adjacent levels is still high, even implanted with the Dynesys system stabilizing the spine [18,21,24]. These studies also indicate that the high stiffness of the Dynesys system itself may be a major contributing factor to disc stress. Our previous study implanted with the Dynesys system [23] demonstrated that reducing the preload on the cord can alleviate stress of the disc and improve ROM at the adjacent levels during flexion. In a subsequent study, we changed the stiffness of the spacers by altering their diameter [24], and showed that spacers with smaller diameters reduce disc stress and ROM at adjacent levels during extension. In the present study, we used FE models to compare Dynesys systems with and without preload of the cord, using either standard (6.0 mm) or smaller (4.8 mm) diameter spacers to determine whether changing the preload on the cord and the stiffness of the spacer can reduce stress concentration at adjacent levels.

### Results from decompressed spine

In our study, the decompressed spine, showed the greatest increase in ROM at L3-4 from the INT model during extension, followed by flexion, axial rotation and lateral bending. This finding is in a accordance with those of Okawa et al. [36] and Bresnahan et al. [37], but it differs from that of Abumi et al. [38], who observed that facectectomy with division of the supraspinous and interspinous ligaments affected flexion stability, but not extension and lateral bending. The in vitro studies from Niosi et al. [39] and Panjabi et al. [40] using decompressed spine specimens, however, show that the ROM increases for all four moments, further supporting our findings.

The greatest increase in disc stress at L3-4 from the INT model occurred during extension, followed by flexion, axial rotation and lateral bending. This result is in agreement with experimental findings by Rao et al. [41] and Cunningham et al. [42]. Furthermore, an in vitro experiment conducted by Haher et al. [43] demonstrated that with the destruction of the facets, axial loads are transferred to the anterior annulus and anterior longitudinal ligament. This could explain why the disc annulus stress at this level for the facetectomy model increased compared to the INT model.

In agreement with Kiapour et al. [44], the greatest decrease in facet joint contact force from the INT model occurred at L3-4 during extension, followed by axial rotation and lateral bending. We observed that the facet joints sustained more contact force on their right sides during axial rotation and sustained more contact force on their left sides during lateral bending. This finding is in agreement with the results of Kuo et al. [45]. According to the report by Serhan et al. [46], the articular surfaces of the facets come into contact during extension, limiting rotation and increasing the compressive load. The facets eventually contact unilaterally during axial rotation, whereas the joint opens during flexion. Hence, as we observed, there should be no contact force at the bridged level facet joint during flexion, as in the current study.

For L2-3, ROM decreased during flexion and extension, but was nearly the same during axial rotation and lateral bending. Disc stress decreased during flexion, extension and axial rotation and increased slightly during lateral bending. Facet joint contact force decreased during extension and axial rotation and increased slightly during lateral bending. The performance of the DEC model at L2-3 compensated for the instability at L3-4. The changes in ROM were more obvious in the sagittal plane than in the transverse and frontal planes; however, the changes in disc stress and facet contact forces were more obvious during extension and axial rotation. These results are similar to, but not in complete agreement, with the report by Zander et al. [47], where the disc stress adjacent to the decompressed level decreased slightly for forward bending when a two-level laminectomy was performed, but remained the same when a single-level laminectomy was performed. Even so, the facet joint forces adjacent to the decompressed level are barely affected by the extent of decompression. The differences between these two studies may be attributed to the hybrid method used in our study. In addition, the disc adjacent to the decompressed level was not assumed to be degenerate in the study by Zander et al., but it was set as degenerate in our study.

### Spinal models implanted with the dynesys system with cord pretension

For the Dynesys system with cord pretension, ROM at L3-4 for the IntDyWL and DecDyWL models decreased markedly during flexion, extension, axial rotation and lateral bending due to shielding from the cord strain and the stiffness of the spacer. The decompressed model further decreased at the sagittal plane due to increased cord strain [24]. Disc stress at the bridged level decreased during flexion, extension and lateral bending, but it increased obvious during axial rotation due to the effect of cord pretension and diminished resistance to axial rotation caused by the Dynesys system, as mentioned previously by Rohlmann et al. [48]. The decompressed model changed even further. Facet joint contact force at the bridged level for the IntDyWL model increased during flexion, axial torsion and lateral bending but decreased during extension. In contrast, the bridged level facet joint contact force of the decompressed model (DecDyWL) decreased during extension, axial rotation and lateral bending but remained the same during flexion because the facetectomy attenuated the facet load. This supported the findings of Kiapour et al. [44] and Lee et al. [49]. At L2-3, the greatest increase in ROM and disc stress occurred for the IntDyWL model during flexion, followed by lateral bending, extension and axial rotation. The greatest increase in facet contact force occurred for the DecDyWL model during lateral bending, followed by extension. The greatest increase in facet contact force occurred for the IntDyWL model during axial rotation.

These results revealed that the Dynsys system with cord pretension can stabilize the bridged level, even for decompressed spines. However, ROM, disc stress, and facet contact forces at the adjacent cranial level increased, especially for the INT model during flexion and lateral bending. For decompressed spines, which need implants for stabilization, the effects on the adjacent levels were less obvious than for intact spines.

### Spinal models implanted with the dynesys system without cord pretension

For spinal modes implanted with the Dynesys system but without cord pretension, ROM at L3-4 decreased to a lesser extent during flexion and lateral bending, compared with the Dynesys system with cord pretension because the attenuated cord strain preserved the joint motion. The ROM decreased more during extension because there was no cord pretension to overcome the stiffness of the spacer. Moreover, the ROM increased during axial rotation because there was no cord pretension to resist the torque from the axial rotation. This finding is consistent with the results of Panjabi et al. [32] who demonstrated that StabilimaxNZ, a flexible stabilizer without pretension, does not effectively stabilize the spine during axial rotation. Disc stress at the bridged level also decreased to a lesser extent during flexion, and increased to a lesser extent during axial rotation, compared implanted with the Dynesys system with cord pretension. Disc stress decreased to a greater extent during extension and lateral bending, compared implanted with the Dynesys system with cord pretension. The effect of spacer shielding on disc stress and facet joint contact force was more obvious during extension and lateral bending if there was no cord pretension. Hence, facet contact forces at the bridged levels in the DecDyNL and DecDyNL0.8 models decreased more due to shielding by the spacers during extension and lateral bending, but not during axial rotation. Softening the spacer (0.8× diameter) did not affect flexion, compared implanted with the Dynesys system with cord pretension, but it alleviated the shielding effect caused by the standard spacer by slightly increasing the ROM, disc stress, and facet joint contact force during extension and lateral bending. However, softening the spacer provided very little benefit in terms of ROM, disc stress, and facet joint contact force during axial rotation. Removing cord pretension decreased ROM and disc stress at L2-3 during flexion, axial rotation, and lateral bending compared with the IntDyWL and DecDyWL models. However, removing the cord pretension decreased ROM and disc stress at the adjacent levels during extension because there was no cord pretension to overcome the stiffness of the spacer, even for the softer spacers used in the DecDyNL0.8 model. Facet contact force increased during extension and lateral bending, and decreased during axial rotation in comparison with the IntDyWL and DecDyWL models.

These results demonstrate that removing cord pretension and even further softening the spacers can alleviate disease at adjacent levels by preserving segment motion, disc stress, and facet contact forces at the bridged level during flexion and lateral bending, but not during extension. For decompressed spines, implanting the Dynesys system without cord pretension does not limit ROM during axial rotation compare to the intact spine. However, the ROM is still less than that of decompressed spine without the Dynesys system. Nevertheless, by removing cord pretension and even softening the spacers, the ROM, facet contact force and disc stress preserved more at the bridged level and changed less at the adjacent level, compared with Dynesys system with cord pretension and standard size spacers, except during extension.

There are several limitations in this study. The vertebrae were simplified to be homogeneous and muscle group insertion into the spinal column was ignored. However, these simplifications had very little influence on the biomechanical behavior in comparison with in vitro study [22]. There was also no follower preload applied in the FE models, however, others have suggested that follower preloads result in the same trends for ROM [36]. It is also important to consider that the stiffness of the Dynesys spacers will change with temperature. Due to a lack of reliable data about the behavior of spacers at different temperatures, we used the stiffness of the spacers at 25°C. Moreover, our study did not consider the viscoelastic behavior of PCU and PET or the threads of the pedicle screws. Furthermore, the relationship between the cords and their surrounding spacers was neglected. During movement, there should be friction between the cords and spacers. This friction, however, is too small to influence the results.

## Conclusion

This study revealed that laminectomy and facetectomy increase the ROM and disc stress at the bridged level and the Dynesys system constrained the ROM of the decompressed spine. However, the Dynesys system did not preserve joint motion at the bridged level and did not diminish disc stress or facet joint contact force at the adjacent cranial level. Removing cord pretension load improved the ROM, disc stress, and facet joint contact forces at the adjacent levels during flexion and left rotation, but did not improve extension and lateral bending moments. By reducing cord pretension load and softening the spacer stiffness, however, we were able to improve ROM, disc stress, and facet contact force at the adjacent levels for all four moments. In spite of the limited benefit found in the present study, we found that the Dynesys without cord pretension can diminish ROM at adjacent levels, while providing secure stabilization even during axial rotation.

## Competing interests

The authors declare that they have no competing interests.

## Authors’ contributions

SL carried out FE analysis and drafted the manuscript. CL participated in study design and clinical discussion. LY and carried out the FE model validation. CH helped to draft the manuscript. CS carried out biomechanical analysis and participated in coordination. All authors read and approved the final manuscript.

## Pre-publication history

The pre-publication history for this paper can be accessed here:

http://www.biomedcentral.com/1471-2474/14/191/prepub

## References

[B1] Bridwell KH Sedgewick TA O’Brien MF Lenke LG Baldus C The role of fusion and instrumentation in the treatment of degenerative spondylolisthesis with spinal stenosis J Spinal Disord Tech 1993 6 6 461 472 10.1097/00002517-199306060-00001 8130395

[B2] Lee CK Lumbar spinal instability (olisthesis) after extensive posterior spinal decompression Spine 1983 8 4 429 433 10.1097/00007632-198305000-00014 6635793

[B3] Johnsson KE Willner S Johnsson K Postoperative instability after decompression for lumbar spinal stenosis Spine 1986 11 2 107 110 10.1097/00007632-198603000-00001 3704799

[B4] Lee CK Accelerated degeneration of the segment adjacent to a lumbar fusion Spine 1988 13 3 375 377 10.1097/00007632-198803000-00029 3388124

[B5] Park P Garton HJ Gala VC Hoff JT McGillicuddy JE Adjacent segment disease after lumbar or lumbosacral fusion: review of the literature Spine 2004 29 17 1938 1944 10.1097/01.brs.0000137069.88904.03 15534420

[B6] Schulte TL Hurschler C Haversath M Liljenqvist U Bullmann V Filler TJ Osada N Fallenberg EM Hackenberg L The effect of dynamic, semi-rigid implants on the range of motion of lumbar motion segments after decompression Eur Spine J 2008 17 8 1057 1065 10.1007/s00586-008-0667-0 18493802 PMC2518758

[B7] Mulholland RC Sengupta DK Rationale, principles and experimental evaluation of the concept of soft stabilization Eur Spine J 2002 11 2 S198 S205 12384745 10.1007/s00586-002-0422-x PMC3611578

[B8] Dubois G de Germay B Schaerer NS Fennema P Szalski M, Gunzburg R, Pope MH Dynamic neutralization: a new concept for restabilization of the spine Lumbar segmental instability 1999 Philadelphia: Lippincott Williams & Wilkins 233 240

[B9] Schaeren S Broger I Jeanneret B Minimum four-year follow-up of spinal stenosis with degenerative spondylolisthesis treated with decompression and dynamic stabilization Spine 2008 33 18 E636 E642 10.1097/BRS.0b013e31817d2435 18708915

[B10] Schnake KJ Schaeren S Jeanneret B Dynamic stabilization in addition to decompression for lumbar spinal stenosis with degenerative spondylolisthesis Spine 2006 31 4 442 449 10.1097/01.brs.0000200092.49001.6e 16481955

[B11] Silvestre MD Lolli F Bakaloudis G Parisini P Dynamic stabilization for degenerative lumbar scoliosis in elderly patients Spine 2010 35 2 227 234 10.1097/BRS.0b013e3181bd3be6 20081518

[B12] Welch WC Cheng BC Awad TE Davis R Maxwell JH Delamarter R Wingate JK Sherman J Macenski MM Clinical outcomes of the Dynesys dynamic neutralization system: 1-year preliminary results Neurosurg Focus 2007 22 1 E8 17608342 10.3171/foc.2007.22.1.8

[B13] Wurgler-Hauri CC Kalbarczyk A Wiesli M Landolt H Fandino J Dynamic neutralization of the lumbar spine after microsurgical decompression in acquired lumbar spinal stenosis and segmental instability Spine 2008 33 3 E66 E72 10.1097/BRS.0b013e31816245c0 18303447

[B14] Kumar A Beastall J Hughes J Karadimas EJ Nicol M Smith F Wardlaw D Disc changes in the bridged and adjacent segments after Dynesys dynamic stabilization system after two years Spine 2008 33 26 2909 2914 10.1097/BRS.0b013e31818bdca7 19092623

[B15] Cakir B Carazzo C Schmidt R Mattes T Reichel H Kafer W Adjacent segment mobility after rigid and semi-rigid instrumentation of the lumbar spine Spine 2009 34 12 1287 1291 10.1097/BRS.0b013e3181a136ab 19455004

[B16] Kim CH Chung CK Jahng TA Comparisons of outcomes after single or multilevel dynamic stabilization: effects on adjacent segment J Spinal Disord Tech 2011 24 1 60 67 10.1097/BSD.0b013e3181d4eb44 21270627

[B17] Castellvi A Huang H Vestgaarden T Saigal S Clabeaux DH Pienkowski D Stress reduction in adjacent level discs via dynamic instrumentation: a finite element analysis SAS Journal 2007 1 2 74 81 10.1016/S1935-9810(07)70050-6 PMC4365575 25802582

[B18] Zander T Rohlmann A Burra NK Bergmann G Effect of a posterior dynamic implant adjacent to a rigid spinal fixator Clin Biomech 2006 21 8 767 774 10.1016/j.clinbiomech.2006.04.001 16750875

[B19] Strube P Tohtz S Hoff E Gross C Perka C Putzier Dynamic stabilization adjacent to single-level fusion. Part I. Biomechanical effects on lumbar spinal motion Eur Spine J 2010 19 12 2171 2180 10.1007/s00586-010-1549-9 20683625 PMC2997204

[B20] Schmoelz W Huber JF Nydegger T Claes L Wilke HJ Dynamic stabilization of the lumbar spine and its effects on adjacent segments: an in vitro experiment J Spinal Disord Tech 2003 16 4 418 423 10.1097/00024720-200308000-00015 12902959

[B21] Shin DS Lee K Kim D Biomechanical study of lumbar spine with dynamic stabilization device using finite element method Computer-Aided Design 2007 39 559 567 10.1016/j.cad.2007.03.005

[B22] Zhang QH Teo EC Effect of Dynamic stabilization device stiffness on disc loading under compression IFMBE Proceedings 2008 19 119 122 10.1007/978-3-540-79039-6_32

[B23] Liu CL Zhong ZC Hsu HW Shih SL Wang ST Hung C Chen CS Effect of the cord pretension of the Dynesys dynamic stabilization system on the biomechanics of the lumbar spine Eur Spine J 2011 20 11 1850 1858 10.1007/s00586-011-1817-3 21523456 PMC3207341

[B24] Shih SL Chen CS Lin HM Huang LY Liu CL Huang CH Cheng CK Effect of spacer diameter of the Dynesys dynamic stabilization system on the biomechanics of the lumbar spine: a finite element analysis J Spinal Disord Tech 2012 25 5 E140 E149 22744611 10.1097/BSD.0b013e31824e5e10

[B25] Zhong ZC Chen SH Hung C Load- and displacement controlled finite element analyses on fusion and non-fusion spinal implants Proc Inst Mech Eng H 2009 223 2 143 157 10.1243/09544119JEIM476 19278192

[B26] Chen SH Zhong ZC Chen CS Chen WJ Hung C Biomechanical comparison between lumbar disc arthroplasty and fusion Med Eng Phys 2009 31 2 244 253 10.1016/j.medengphy.2008.07.007 18760654

[B27] Liu CL Zhong ZC Shih SL Hung C Lee YE Chen CS Influence of Dynesys system screw profile on adjacent segment and screw J Spinal Disord Tech 2010 23 6 410 417 10.1097/BSD.0b013e3181b63d89 20683426

[B28] Umehara S Tadano S Abumi K Katagiri K Kaneda K Ukai T Effects of degeneration on the elastic modulus distribution in the lumbar intervertebral disc Spine 1996 21 7 811 819 10.1097/00007632-199604010-00007 8779011

[B29] Rohlmann A Zander T Bergmann G Effect of total disc replacement with ProDisc on the biomechanical behavior of the lumbar spine Spine 2005 30 7 738 743 10.1097/01.brs.0000157413.72276.c4 15803074

[B30] Schmidt H Heuer F Simon U Kettler A Rohlmann A Claes L Wilke HJ Application of a new calibration method for a three-dimensional finite element model of a human lumbar annulus fibrosus Clin Biomech 2006 21 4 337 344 10.1016/j.clinbiomech.2005.12.001 16439042

[B31] Kettler A Rohlmann F Ring C Mack C Wilke HJ Do early stages of lumbar intervertebral disc degeneration really cause instability? Evaluation of an in vitro database Eur Spine J 2011 20 4 578 584 10.1007/s00586-010-1635-z 21125299 PMC3065606

[B32] Ruberte LM Natarajan RN Andersson GB Influence of single-level lumbar degenerative disc disease on the behavior of the adjacent segments–a finite element model study J Biomech 2009 42 3 341 348 10.1016/j.jbiomech.2008.11.024 19136113

[B33] McMillan DW McNally DS Garbutt G Adams MA Stress distributions inside intervertebral discs: The validity of experimental stress profilometry Proc Inst Mech Eng H 1996 210 2 81 87 8688120 10.1243/PIME_PROC_1996_210_396_02

[B34] Yamamoto I Panjabi MM Crisco T Oxland T Three-D Moments of the Whole Lumbar Spoie and Lumbosacral Joint Spine 1989 14 11 1256 1260 10.1097/00007632-198911000-00020 2603060

[B35] Panjabi MM Hybrid multidirectional test method to evaluate spinal adjacent - level effects Clin Biomech 2007 22 3 257 265 10.1016/j.clinbiomech.2006.08.006 17196720

[B36] Okawa A Shinomiya K Takakuda K Nakai O A cadaveric study on the stability of lumbar segment after partial laminotomy and facetectomy with intact posterior ligaments J Spinal Disord 1996 9 6 518 526 8976493

[B37] Bresnahan L Ogden AT Natarajan RN Fessler RG A biomechanical evaluation of graded posterior element removal for treatment of lumbar stenosis: comparison of a minimally invasive approach with two standard laminectomy techniques Spine 2009 34 1 17 23 10.1097/BRS.0b013e318191438b 19127157

[B38] Abumi K Panjabi MM Kramer KM Duranceu J Oxland T Crisco JJ Biomechanical evaluation of lumbar spine stability after graded facetectomies Spine 1990 15 11 1142 1147 10.1097/00007632-199011010-00011 2267608

[B39] Niosi CA Zhu QA Wilson DC Keynan O Wilson DR Oxland TR Biomechanical characterization of the three-dimensional kinematic behaviour of the Dynesys dynamic stabilization system: an in vitro study Eur Spine J 2006 15 6 913 922 10.1007/s00586-005-0948-9 16217663 PMC3489456

[B40] Panjabi MM Henderson G James Y Timm P StabilimaxNZ versus simulated fusion: evaluation of adjacent-Level effects Eur Spine J 2007 16 12 2159 2165 10.1007/s00586-007-0444-5 17924151 PMC2140135

[B41] Rao RD Wang M Singhal P Intradiscal pressure and kinematic behavior of lumbar spine after bilateral laminotomy and laminectomy Spine J 2002 2 5 320 326 10.1016/S1529-9430(02)00402-3 14589462

[B42] Cunningham BW Kotani Y McNulty PS Cappuccino A McAfee PC The effect of spinal destabilization and instrumentation on lumbar intradiscal pressure: an in vitro biomechanical analysis Spine 1997 22 22 2655 2263 10.1097/00007632-199711150-00014 9399452

[B43] Haher TR O'Brien M Dryer JW Nucci R Zipnick R Leone DJ **The role of the lumbar facet joints in spinal stability**. Identification of alternative paths of loading Spine 1994 19 23 2667 2671 7899961

[B44] Kiapour A Ambati D Hoy RW Goel VK Effect of graded facetectomy on biomechanics of Dynesys dynamic stabilization system Spine 2012 37 10 E581 E589 10.1097/BRS.0b013e3182463775 22198353

[B45] Kuo CS Hu HT Lin RM Huang KY Lin PC Zhong ZC Hseih ML Biomechanical analysis of the lumbar spine on facet joint force and intradiscal pressure - a finite element study BMC Musculoskelet Disord 2010 11 151 10.1186/1471-2474-11-151 20602783 PMC2913991

[B46] Serhan HA Varnavas G Dooris AP Patwadhan A Tzermiadianos M Biomechanics of the posterior lumbar articulating elements Neurosurg Focus 2007 22 1 E1 17608330 10.3171/foc.2007.22.1.1

[B47] Zander T Rohlmann A Klockner C Bergmann G Influence of graded facetectomy and laminectomy on spinal biomechanics Eur Spine J 2003 12 427 434 10.1007/s00586-003-0540-0 12720068 PMC3467787

[B48] Rohlmann A Boustani HN Bergmann G Zander T Effect of a pedicle-screw- based motion preservation system on lumbar spine biomechanics: a probabilistic finite element study with subsequent sensitivity analysis J Biomech 2010 43 2963 2969 10.1016/j.jbiomech.2010.07.018 20696430

[B49] Lee KK Teo EC Qiu TX Yang K Effect of facetectomy on lumbar spinal stability under sagittal plane loadings Spine 2004 29 15 1624 1631 10.1097/01.BRS.0000132650.24437.15 15284506

